# On the Link between Perceived Parental Rearing Behaviors and Self-conscious Emotions in Adolescents

**DOI:** 10.1007/s10826-017-0695-7

**Published:** 2017-03-10

**Authors:** Cor Meesters, Peter Muris, Pauline Dibbets, Maaike Cima, Lotte Lemmens

**Affiliations:** 1 Clinical Psychological Science, Maastricht University, Maastricht, The Netherlands; 2 Department Developmental Psychopathology, Radboud University, Brain Science Institute, Nijmegen, The Netherlands

**Keywords:** Parental rearing, Self-conscious emotions, Guilt, Shame, Adolescents

## Abstract

This study examined relationships between the self-conscious emotions of guilt and shame in both clinical (*N* = 104) and non-clinical (*N* = 477) (young) adolescents aged 11–18 years, who completed a questionnaire to assess perceived parental rearing behaviors (EMBU-C) and a scenario-based instrument to measure proneness to guilt and shame (SCEMAS). Results indicated that parental rearing dimensions were positively related to self-conscious emotions. Regarding the non-clinical sample, both favourable (emotional warmth) and unfavourable (rejection) paternal and maternal rearing dimensions were significant correlates of guilt- and shame-proneness. The results for the clinical sample were less conclusive: only maternal emotional warmth and rejection were found to be significantly associated with guilt and shame. Interestingly, no associations between any of the paternal rearing dimensions and self-conscious emotions emerged. Taken together, these results are in keeping with the notion that parental rearing factors are involved in the development of both adaptive and maladaptive self-conscious emotions in adolescents.

## Introduction

Research on moral emotions has mainly focused on the negatively valenced self-conscious emotions of shame and guilt. Although these two emotions are often regarded as equivalent, research has indicated that there are reasons to assume that they are quite different (e.g., Tangney et al. 2007; Tangney and Tracy 2012). This distinction is clearly expressed by Lewis (1971) who noted that guilt is concerned with negative feelings about a specific behavior or action undertaken by a person (“I did *that bad thing*”), whereas shame pertains to negative feelings about the global self (“*I* did that bad thing”). From this it follows that guilt is concerned with feelings of remorse and regret about one’s behavior, thereby prompting the person to undertake reparative actions (Tangney and Dearing 2002), whereas shame is accompanied by feelings of worthlessness and incompetence, and a wish to escape and withdraw from social contact (Tangney et al. 2007; Tracy and Robins 2004).

Given these functional differences between shame and guilt, it comes as no surprise that shame is considered as a less adaptive emotion than guilt (e.g., Pineless et al. 2006; Tangney and Dearing 2002; Tangney et al. 1992). For example, research on the association between both types of self-conscious emotions and psychopathology has demonstrated that shame is indeed positively associated with a broad range of psychological problems, including anger and externalization (e.g., Tangney et al. 1996), anxiety disorders (e.g., Fergus et al. 2010; Gilbert 2000), posttraumatic stress disorder (Andrews et al. 2000), depression (e.g., Thompson and Berenbaum 2006), eating disorders (Troop et al. 2008), personality disorders (Brown et al. 2009; Schoenleber and Berenbaum 2010), and alcohol and drug abuse (e.g., Dearing et al. 2005). The link between guilt and psychopathology has been found to be more ambiguous. There is some research showing that guilt is also positively linked to psychological problems, but this is mainly the case when this emotion is experienced in a ruminative way or merged with feelings of shame (Tangney and Tracy 2012). There is accumulating evidence showing that guilt in itself serves an adaptive interpersonal function, as it stimulates restorative actions in case of moral transgressions, thereby promoting the development of empathy and conscience and reducing aggression and other externalizing problems (Baumeister et al. 1995; Kochanska et al. 2012; Stuewig et al. 2010; Tangney et al. 1996).

Although the majority of research on the relationship between self-conscious emotions and psychopathology has been conducted in adult populations (for an overview, see Tangney and Tracy 2012), the number of studies on the link between shame and guilt and psychological problems in children and adolescents is steadily increasing. A recent review by Muris and Meesters (2014) including 22 empirical studies demonstrated that shame and guilt in youths were in a similar way related to psychopathology as found in studies with adults. That is, in general shame was positively linked to both internalizing and externalizing symptoms, while guilt only appeared to be negatively correlated with externalizing problems.

Muris and Meesters (2014) also noted that the dysregulation of self-conscious emotions already have their roots during childhood and that various factors, both innate and contextual in nature, contribute to individual differences in shame and guilt proneness. Parenting behaviors constitute a relevant contextual factor in this regard. That is, research suggests that negative parental rearing behaviors play a role in children’s proneness to experience shame. More specific, data from both retrospective studies with adults and cross-sectional and longitudinal studies with children and adolescents have shown that shame is positively connected with an authoritarian parenting style (Mills 2003), denigration, indifference, rejection, and abandonment of parents (Claesson and Sohlberg 2002; Gilbert et al. 2003; Han and Kim 2012; Harvey et al. 1997), parental use of conditional positive regard (Assor and Tal 2012), parentification (Wells and Jones 2000), negative parental evaluative behavior (Alessandri and Lewis 1993), and disparagement and shaming (Gilbert et al. 1996; Mills et al. 2010).

Evidence on the possible role of parental rearing in the development of guilt in youths is rather meager. A study by Zahn-Waxler et al. (1990) has demonstrated that children of depressed parents, who often engage in negative parenting (Susman et al. 1985) more often displayed deviant and distorted expressions of guilt (e.g., guilt responses being fused with shame) and as a consequence frequently failed to repair social transgressions than children of healthy, non-depressed parents. In her review article on the development of morality Eisenberg (2000) emphasized that children develop adaptive guilt reactions when growing up with parents who induce morality in their children in case of a transgression, especially when such inductions are carried out in an affectionate way (see also Scarnier et al. 2009). On the other hand, when parents are not warm and caring and are inclined to induce feelings of guilt in a negative way—which often applies to clinically depressed parents (Rakow et al. 2009, 2011)—the development of guilt may go wrong (Stuewig and McCloskey 2005).

To summarize, research has demonstrated that shame and guilt are related in a theoretically meaningful way with various types of psychopathological symptoms in youths. There is also evidence indicating that parenting behaviors contribute to the development of these self-conscious emotions, although such research is relatively sparse, in particular regarding guilt. With this in mind, the present study was set up in order to examine the relation between both positive (i.e., emotional warmth) and negative (i.e., rejection) perceived parental rearing and the self-conscious emotions of guilt and shame in two different samples of children and adolescents. The first sample consisted of non-clinical adolescents aged 11–18 years, whereas the second sample concerned youths aged 13–18 years, who were referred to a clinical institution. All participants completed the child version of the EMBU (My Memories of Upbringing; Muris et al. 2003), a questionnaire to assess perceived parental rearing behaviors, and the SCEMAS (Self-Conscious Emotions: Maladaptive and Adaptive Scales; Stegge and Ferguson 1994), a scenario-based instrument for assessing proneness to guilt and shame. It was investigated whether (1) parental rejection is linked to heightened levels of shame proneness, (2) parental emotional warmth is connected with higher levels of guilt proneness, and (3) whether the association between parental rearing behaviors and proneness to guilt and shame is different for clinical and non-clinical youths.

## Method

### Participants

This study was carried out in two different samples of (young) adolescents. The first sample was recruited from four secondary schools in the south-eastern part of the Netherlands and the eastern part of Belgium. In total, 477 youngsters (224 boys and 253 girls) were given permission to participate, yielding a response rate of 69%. The mean age of the participants was 13.88 years (*SD* = 1.78; range 11–18 years). More than 90% of the participants were from original Dutch or Belgian origin, and based on the school records it was estimated that 22.4 had a low, 44.7 a middle, and 32.9% a high socio-economic status.

The second sample consisted of 104 youths (69 boys and 35 girls) who were referred to the Child Care and Protection Board (response rate: 22%). Their mean age was 15.53 years (*SD* = 1.16; range 13–18 years). Adolescents were predominantly characterized by externalizing problem behaviors, and all of them had come into contact with the police or the law, e.g., due to vandalism, aggressive behavior, theft, or possession of and/or dealing drugs. Ninety-five percent of the participants were Caucasian, and 52 adolescents (50%) came from broken families. Some youths (5.7%) no longer attended school, but the great majority of them received (lower) preparatory secondary vocational education (71.1%). Based on the level of education of the parents, estimations of low, middle, and high socio-economic status of the participants were 51.8, 33.9, and 14.3%, respectively.

### Procedure

All eligible adolescents from the regular secondary schools and their caregivers received a letter with information about the study together with a consent form. Participants were tested during regular classes at school in the presence of a research assistant and a teacher to ensure confidentiality and to provide assistance when needed.

After referral to the Child Care and Protection Board, parents and caregivers were asked for permission to participate by sending them an information letter about the study together with an informed consent form. All clinically referred adolescents completed the set of questionnaires during their visit of the Child Care Protection Board individually in presence of a research assistant. This study was approved by the Ethical Committee of Psychology of Maastricht University.

### Measures

#### Perceived parental rearing

The EMBU-C (Castro et al. 1993) is a child version of the EMBU (“Egna Beträffande Uppfostran-My memories of upbringing; Perris et al. 1980), an originally Swedish instrument to assess a person’s view of his/her parents’ rearing behaviors. For the purpose of the present study two subscales of a modified version of the EMBU-C (Muris et al. 2003) were used, that is, emotional warmth (10 items; e.g., “Your parents listen to you and consider your opinion”), and rejection (10 items; e.g., “Your parents treat you unfairly”). For each EMBU-C item, participants first rated their father’s rearing behavior and then their mother’s rearing behavior on a 4-point Likert scale (1 = “No, never, 2 = “Yes, but seldom, 3 = “Yes, often”, 4 = “Yes, most of the time”). Previous research has demonstrated that the EMBU-C is a reliable and valid instrument for the assessment of children and adolescents’ perceptions of parental rearing behaviors (Castro et al. 1993; Muris et al. 2003; Roelofs et al. 2006).

#### Self-conscious emotions

The SCEMAS (Self-Conscious Emotions: Maladaptive and Adaptive Scales; Stegge and Ferguson 1994) is designed to measure children’s proneness to experience self-conscious emotions. The SCEMAS consists of 13 situations in which the child is described as the protagonist in interaction with peers of the same sex. Eight of these scenarios originated from the Child-Child Reaction and Attribution Survey (C-CARS; Stegge and Ferguson 1990) and present situations in which the child protagonist clearly failed to achieve a task or did something wrong in front of others, either deliberately or at least manageably. An example of such an unambiguous situation is: “You are outside riding on your bike. You are not paying proper attention where you are going, and by accident you ride right over the toy of a little boy. The little boy sees the broken toy and starts to cry. You have seen the broken toy and the crying boy, but you keep riding on your bike towards home”. The story is followed by a list of possible reactions, and the child is requested to rate to what extent each response is applicable to him or her, using a 5-point Likert scale ranging from 0 (not at all) to 4 (very much). The options relate to the self-conscious emotions of shame (“You feel like a bad person for leaving the boy and not helping him”), guilt (“You feel sorry”), and ruminative guilt (“You feel guilty for a very long time”). In the remaining 5 situations, the child protagonist’s behavior was almost always uncontrollable and definitely had negative consequences for another person; nevertheless, children could have positive feelings about their accomplishments. This way, a more ambiguous situation was created, for example, “A very popular boy in your class is giving a birthday party. He has a lot of friends, yet he is only allowed to invite a few children because it is really going to be a special party. You are invited for the party and you are really excited. You are telling your best friend about it, but then you notice that he is sad as he is not invited”. As with the unambiguous scenarios, the responses to these ambiguous scenarios concern shame (“You think: I am not such a good friend after all; I only think of myself”), guilt (“You feel bad about yourself because you didn’t take your friend’s feelings into account”), and ruminative guilt (“You worry a long time about how bad your friend should feel because he is not invited”). The inclusion of both unambiguous and ambiguous situations is based on the idea that it is more adaptive to experience guilt and shame in the former than in the latter situation. In the current study a number of scores were created by averaging the appropriate ratings across targeted scenarios (minimum score = 0; maximum score = 4), yielding the following SCEMAS subscales: total guilt (13 items), guilt-unambiguous (8 items), guilt-ambiguous (5 items), total shame (13 items), shame-unambiguous (8 items), shame-ambiguous (5 items), and ruminative guilt (13 items). Previous research has indicated that the SCEMAS is a reliable and valid measure of proneness to guilt and shame in youths (Ferguson et al. 2000; Muris et al. 2014).

### Data Analyses

SPSS (version 21) was used to calculate descriptive statistics, reliability coefficients, and to carry out *t*-tests and correlational analysis. To examine the relation between perceived parental rearing and self-conscious emotions, hierarchical regression analyses were conducted in which various types of self-conscious emotions were predicted from the two EMBU-C dimensions for mother and father. In these analyses, both EMBU-C dimensions were entered simultaneously into the equation in order to investigate their relative contribution to levels of self-conscious emotions. To control for the possible effect of age and gender, these variables were always entered on step 1. Further, since earlier research has shown that guilt and shame have considerable overlap (e.g., Tangney 1996), all regressions were performed for a second time. So, in the analyses with shame scores as the dependent variable, guilt was added as a covariate, whereas in the analyses in which guilt was the dependent variable, shame was included as a covariate.

## Results

Before presenting the main findings of the present study, a number of general results will be discussed (see Tables 1 and 2). First, the SCEMAS and the EMBU-C proved to be reliable instruments in both the clinical and non-clinical sample of youths: most Cronbach’s alphas being > .60 (range .61–.87). The only exception was the insufficient alpha (.56) for the SCEMAS subscale ambiguous guilt regarding the clinical sample. Note however, that this scale only comprised 5 items. Second, both clinical and non-clinical adolescents reported higher levels of guilt than of shame [paired *t*(101) = 9.06, *p* < .001, and paired *t*(474) = 14.5, *p* < .001, respectively], and ruminative guilt [paired *t*(101) = 10.04, *p* < .001, and paired *t*(474) = 16.47, *p* < .001, respectively]. Furthermore, as one would expect, in both samples guilt- and shame responses to unambiguous scenarios were significantly higher than guilt- and shame responses to ambiguous scenarios [guilt: paired *t*(101) = 13.07, *p* < .001, and paired *t*(474) = 33.9, *p* < .001, respectively; shame: paired *t*(101) = 9.72, *p* < .001, and paired *t*(474) = 23.43, *p* < .001, respectively]. Third, in both samples significant gender differences were found for most SCEMAS scales: with the exception of ambiguous guilt girls exhibited higher levels of self-conscious emotions than boys [non-clinical sample: *t*(473)s ≥ 3.03, *p*s < .01; clinical sample: *t*(100)s ≥ 2.71, *p*s < .01]. Fourth, clinical youths reported significantly lower levels of self-conscious emotions than non-clinical youths [all *t*(575)s ≥ 3.57, all *p*s < .001]. Fifth, clinical adolescents reported lower levels of emotional warmth of father [*t*(550) = −2.57, *p* < .05] as compared to their non-clinical counterparts. Sixth and final, in both samples the various SCEMAS scales appeared to be substantially correlated (*r*s varying between .55 and .88, all *p*s < .001), indicating that several dimensions of self-conscious emotions clearly show overlap.

**Table 1:** Descriptive statistics for the SCEMAS and the EMBU-C for adolescents from regular secondary schools

	Total sample (*N* = 477)	Boys (*n* = 224)	Girls (*n* = 253)	Cronbach’s α	*r* age
SCEMAS					
Guilt	1.81 (.74)_1_	1.65 (.77)	1.95 (.68)_c_	.85	−.27**
Guilt-unambiguous	2.25 (.89)_a_	2.03 (.92)	2.44 (.81)_c_	.83	−.23**
Guilt-ambiguous	1.11 (.70)_b_	1.05 (.71)	1.17 (.68)	.64	−.27**
Shame	1.54 (.75)_2_	1.36 (.74)	1.70 (.73)_c_	.85	−.27**
Shame-unambiguous	1.86 (.89)_a_	1.63 (.88)	2.06 (.85)_c_	.83	−.26**
Shame-ambiguous	1.04 (.74)_b_	.93 (.69)	1.14 (.77)_c_	.68	−.21**
Ruminative guilt	1.51 (.75)_2_	1.36 (.76)	1.65 (.72)_c_	.85	−.24**
EMBU-C mother					
Rejection	15.88 (4.76)	16.22 (4.74)	15.59 (4.77)	.83	.21**
Emotional warmth	30.85 (5.61)	30.26 (5.41)	31.35 (5.75)	.86	−.26**
EMBU-C father					
Rejection	15.71 (4.89)	16.13 (4.90)	15.35 (4.87)	.84	.24**
Emotional warmth	29.88 (5.95)	29.62 (5.59)	30.11 (6.25)	.87	−.29**

For the total sample, within-column means not sharing similar subscript digits or letters differ at *p* < .001

Subscript c indicating significant gender difference at *p* < .05

*SCEMAS* Self-conscious Emotions Maladaptive and Adaptive Scales*, EMBU-C* Egna Minnen Beträffande Uppfostran/My memories of upbringing-Child version

*p < .01; **p < .001

**Table 2:** Descriptive statistics for the SCEMAS and the EMBU-C for clinically referred adolescents

	Total sample (*N* = 104)	Boys (*n* = 69)	Girls (*n* = 35)	Cronbach’s α	*r* age
SCEMAS					
Guilt	1.39 (.72)_1_	1.26 (.68)	1.65 (.73)_c_	.85	−.02
Guilt-unambiguous	1.74 (.89)_a_	1.56 (.87)	2.08 (.83)_c_	.84	−.01
Guilt-ambiguous	.85 (.60)_b_	.78 (.53)	.97 (.72)	.56	−.04
Shame	1.06 (.64)_2_	.90 (.58)	1.37 (.65)_c_	.82	−.12
Shame-unambiguous	1.34 (.83)_a_	1.16 (.78)	1.69 (.81)_c_	.81	−.13
Shame-ambiguous	.60 (.58)_b_	.47 (.46)	.86 (.70)_c_	.61	−.04
Ruminative guilt	1.06 (.65)_2_	.91 (.56)	1.33 (.72)_c_	.82	−.05
EMBU-C mother					
Rejection	15.86 (4.33)	15.76 (4.41)	16.06 (4.22)	.81	.08
Emotional warmth	30.32 (5.77)	29.91 (5.78)	31.09 (5.76)	.87	−.25*
EMBU-C father					
Rejection	16.44 (5.50)	16.11 (5.21)	17.13 (6.09)	.87	−.12
Emotional warmth	28.15 (6.29)	28.40 (5.90)	27.66 (7.09)	.87	−.10

For the total sample, within column means not sharing similar subscript digits or letters differ at *p* < .001

Subscript c indicating significant gender difference at *p* < .05

*SCEMAS* Self-conscious Emotions Maladaptive and Adaptive Scales, *EMBU-C* Egna Minnen Beträffande Uppfostran/My memories of upbringing-Child version

*p < .05

### Perceived Parental Rearing and Self-conscious Emotions

Tables 3 and 4 present correlations (corrected for age and gender) between perceived parental rearing behaviors and self-conscious emotions for non-clinical and clinical youths, respectively. In non-clinical adolescents emotional warmth, especially of the mother, was positively linked to self-conscious emotions, whereas only paternal rejection appeared to be positively related to (ambiguous) shame and ruminative guilt. With respect to clinical youths, only maternal emotional warmth was found to be positively correlated with guilt- and shame-proneness, whereas all other relationships were non-significant. It is interesting to note, that correlations between parental rearing dimensions and self-conscious emotions were generally weaker in clinical adolescents as compared to adolescents from regular secondary schools and that both paternal emotional warmth and rejection were found to be unrelated to self-conscious emotions.

**Table 3:** Pearson product-moment correlations (corrected for age and gender) between EMBU-C scales and SCEMAS scales for adolescents from regular secondary schools (*N* = 477)

	EMBU-C mother		EMBU-C father	
	Rejection	Emotional warmth	Rejection	Emotional warmth
SCEMAS				
Guilt	−.06	.23***	−.01	.18***
Guilt-unambiguous	−.09	.26***	−.04	.22***
Guilt-ambiguous	.01	.10*	.08	.05
Shame	.03	.13*	.13**	.06
Shame-unambiguous	−.01	.17**	.08	.10*
Shame-ambiguous	.09	.03	.19***	-.03
Ruminative guilt	.05	.11*	.11*	.07

*SCEMAS* Self-conscious Emotions Maladaptive and Adaptive Scales, *EMBU-C* Egna Minnen Beträffande Uppfostran/My

memories of upbringing-Child version

*p *<* .05; **p < .01; ***p < .001

**Table 4:** Pearson product-moment correlations (corrected for age and gender) between EMBU-C scales and SCEMAS scales for clinically referred adolescents (*N* = 104)

	EMBU-C mother		EMBU-C father	
	Rejection	Emotional warmth	Rejection	Emotional warmth
SCEMAS				
Guilt	−.01	.24*	−.15	.16
Guilt-unambiguous	−.04	.25*	−.18	.14
Guilt-ambiguous	.07	.16	−.04	.17
Shame	.04	.27**	−.07	.11
Shame-unambiguous	−.02	.27**	−.07	.07
Shame-ambiguous	.16	.13	−.02	.14
Ruminative guilt	.11	.18	.03	.06

*SCEMAS* Self-conscious Emotions Maladaptive and Adaptive Scales, *EMBU-C* Egna Minnen Beträffande Uppfostran/My

memories of upbringing-Child version

*p *<* .05; **p < .01

### Unique Contributions of Perceived Parental Rearing to Self-conscious Emotions

Multiple linear regression analyses were performed to examine whether perceived parental rearing dimensions account for significant portions of the variance of several types of self-conscious emotions. These regression analyses were carried out for the clinical and non-clinical sample, separately. Regarding non-clinical adolescents (see Table 5), significant and positive β values were found between emotional warmth and rejection of both parents on the one hand and several dimensions of self-conscious emotions on the other hand. These findings indicate that higher levels of parental emotional warmth as well as parental rejection were accompanied by higher levels of (un)ambiguous guilt, (un)ambiguous shame, and ruminative guilt. It should be mentioned that percentages of explained variance in self-conscious emotions scores were rather modest (range: 7–16%).

**Table 5:** Main results of the multiple linear regression analysis with SCEMAS scales as dependent variables and EMBU-C scales as predictor variables in adolescents from regular secondary schools (*N* = 477)

	EMBU-C mother			EMBU-C father		
	Rejection	Emotional warmth	*R* ^2^	Rejection	Emotional warmth	*R* ^2^
SCEMAS						
Guilt	.12*	.31***	.15***	.19**	.31***	.15***
Guilt-unambiguous	.12*	.33***	.16***	.17**	.33***	.15***
Guilt-ambiguous	.10	.16**	.09***	.18**	.17**	.09***
Shame	.17**	.24***	.13***	.28***	.24***	.15***
Shame-unambiguous	.14*	.25***	.13***	.23***	.25***	.14***
Shame-ambiguous	.17**	.13*	.07***	.29***	.16*	.10***
Ruminative guilt	.18**	.21***	.10***	.26***	.24***	.12***

Standardized β values as well as percentage of explained variance are displayed

*SCEMAS* Self-conscious Emotions Maladaptive and Adaptive Scales, *EMBU-C* Egna Minnen Beträffande Uppfostran/My memories of upbringing-Child version. All regression analyses were controlled for age and gender

*p *<* .05; **p < .01; ***p < .001

Table 6 presents the results of the regression analyses for the clinical sample. As can be seen, emotional warmth of mother was positively associated with all self-conscious emotion variables. In addition, rejection by mother was also positively linked with ambiguous shame and ruminative guilt. Remarkably, no significant relation between paternal rearing and any of the self-conscious emotions was found.

**Table 6:** Main results of the multiple linear regression analysis with SCEMAS scales as dependent variables and EMBU-C scales as predictor variables in clinically referred adolescents (*N* = 104)

	EMBU-C mother			EMBU-C father		
	Rejection	Emotional warmth	*R* ^2^	Rejection	Emotional warmth	*R* ^2^
SCEMAS						
Guilt	.11	.29*	.13*	−.08	.11	.11*
Guilt-unambiguous	.07	.28*	.14**	−.14	.05	.12*
Guilt-ambiguous	.16	.23*	.07	.08	.22	.06
Shame	.17	.33**	.22***	−.01	.10	.16**
Shame-unambiguous	.10	.31**	.19**	-.04	.04	.13*
Shame-ambiguous	.25*	.23*	.17**	.08	.18	.11*
Ruminative guilt	.21*	.27*	.16**	.09	.11	.13*

Standardized β values as well as percentage of explained variance are displayed

*SCEMAS* Self-conscious Emotions Maladaptive and Adaptive Scales, *EMBU-C* Egna Minnen Beträffande Uppfostran/My memories of upbringing-Child version. All regression analyses were controlled for age and gender

*p *<* .05; **p < .01; ***p < .001

Running all regression analyses for a second time, while controlling for the non-predicted self-conscious emotion, yielded generally the same pattern of outcomes.

## Discussion

The present study investigated the relationship between perceived parental rearing behaviors and the self-conscious emotions of guilt and shame in both clinical and non-clinical (young) adolescents aged 11–18 years. The main results can be summarized as follows. First, with regard to the non-clinical sample, both parental emotional warmth and parental rejection accounted for significant proportions of the variance in guilt- and shame-proneness. More specifically, emotional warmth of both mother and father explained significant portions of the variance in all indices of guilt and shame, whereas rejection by father was found to be more strongly associated to various dimensions of self-conscious emotions than rejection by mother. Note, however, that positive parental rearing (emotional warmth) as well as negative parental rearing (rejection) are found to be positively related with self-conscious emotions. Although the finding that parental emotional warmth is positively correlated with guilt- and shame-proneness may be considered as counterintuitive, it resembles the results of several other studies. For instance, Belsky et al. (1997) found that children with mothers and fathers who displayed negative parenting behaviors expressed the lowest levels of shame. Further, evidence indicates that favorable rearing behaviors such as responsiveness, care and warmth foster the development of adaptive self-conscious emotions like guilt (see e.g. the work of Kochanska et al. 2005; 2008).

The findings with respect to the sample of clinically referred youths were clearly less convincing. As indicated before, the majority of these youths obviously exhibited externalizing problem behaviors. First of all, results showed that the clinically referred adolescents reported lower levels of self-conscious emotions than adolescents from regular secondary schools. Further, only maternal emotional warmth accounted for significant portions of the variance in various indices of shame and guilt. Interestingly, no associations were found between any of the paternal rearing dimensions and self-conscious emotions. The latter outcome is in line with some previous studies on child and adolescent coping, which showed maternal rearing to be much more associated with coping styles than paternal rearing (e.g., Kliewer et al. 1996; Ruchkin et al. (1999). There is also evidence, however, that negative paternal rearing styles (i.e., paternal rejection and control) do play a role in child and adolescent maladjustment (e.g., Ruchkin et al. 2001; Van Aken et al. 2007; Verhoeven et al. 2012).

It should be noted that in both the clinical and non-clinical sample the strength of observed relationships was rather small. This should not come as a surprise given that parental rearing is not the only factor promoting the development of guilt- and shame-proneness in children and adolescents. For example, research indicates that a child’s rank on the social hierarchy contributes to the experience of self-conscious emotions. More specifically, Gilbert (1997; 2000) has demonstrated that children low in social rank are more inclined to evaluate themselves through other people’s eyes, and as a consequence experience more often self-conscious emotions as guilt and shame. In addition, a factor pertaining to the quality of the parent-child relationship seems also relevant for the emergence of guilt- and shame proneness. Attachment theory (Bowlby 1969, 1973) argues that the establishment of affectional ties with primary caregivers constitutes the fundamental basis for socio-emotional development, that is, secure attachment bonds will lead to a basic trust in the self and in others. In contrast, insecurely attached children have a basic feeling of non-acceptance and mistrust, and therefore are more prone to experience rejection and the self-conscious emotions of guilt and shame (see Lewis 1971). A recent study by Muris et al. (2014) has provided initial evidence for this supposed connection between attachment (in)security and children’s proneness to experience guilt and shame. To be more specific, in a large community sample of youths (*N* = 688) it was found that children who classified themselves as insecurely attached exhibited higher levels of shame and maladaptive forms of guilt as compared to securely attached youngsters.

A few remarks on the present study’s findings are in order. To begin with, in both samples girls experienced higher levels of guilt and shame than boys. This finding is in correspondence with earlier research which also has demonstrated significant gender effects for self-conscious emotions in youths (see a meta-analysis by Else-Quest et al. 2012). Second, the present study used the SCEMAS to measure proneness to guilt and shame in children and adolescents. This instrument resembles the widely used TOSCA-C (Tangney et al. 1990), which is also a scenario-based measure of youths’ propensity to experience self-conscious emotions. However, the SCEMAS permits the discrimination between adjusted (i.e., unambiguous) and maladjusted (i.e., ambiguous) forms of guilt- and shame proneness, which may be an advantage. A recent study by Muris et al. (2014) lends preliminary support for this notion.

Several shortcomings of the current study have to be acknowledged. First, due to its cross-sectional nature, drawing conclusions about causation is impossible. Future prospective research is needed to address issues like how both positive and negative parenting behaviors influence the development of (mal)adaptive self-conscious emotions. A second drawback of the present study has to do with the assessment of parental rearing by means of self-report. In future studies, both observational measures and multiple informants (e.g., parents and teachers) may provide additional valuable information on the association between parental rearing and self-conscious emotions in youths. Third, only 22% of the approached clinically referred youths agreed to participate in the present study. Consequently, the representativeness of the clinical sample may be questioned. Fourth and final, the reliability of some scales used in the current study was found to be modest, and therefore the present findings should be interpreted with caution.

Despite these limitations, however, the results of the present study are generally in line with the accumulated empirical evidence of the relation between parental rearing and self-conscious emotions in youths so far, and clearly justify further investigation.
